# MicroRNA-106b promotes colorectal cancer cell migration and invasion by directly targeting DLC1

**DOI:** 10.1186/s13046-015-0189-7

**Published:** 2015-07-30

**Authors:** Guang-jun Zhang, Jian-shui Li, He Zhou, Hua-xu Xiao, Yu Li, Tong Zhou

**Affiliations:** The Department of Gastrointestinal Surgery, The Affiliated Hospital of North Sichuan Medical College, Nanchong, Sichuan People’s Republic of China; Department of Hepatobiliary Surgery, The Affiliated Hospital of North Sichuan Medical College, Nanchong, Sichuan People’s Republic of China; Institute of Hepatobiliary, Pancreatic and Intestinal Disease, North Sichuan Medical College, Nanchong, Sichuan People’s Republic of China; Department of Pathology, The North Sichuan Medical College, Nanchong, Sichuan People’s Republic of China; Department of Microbiology and Parasitology, North Sichuan Medical College, Nanchong, Sichuan People’s Republic of China

**Keywords:** Colorectal cancer, miR-106b, DLC1, Migration, Invasion, Prognosis

## Abstract

**Background:**

Growing evidence suggests that microRNAs (miRNAs) play an important role in tumor development, progression and metastasis. Aberrant miR-106b expression has been reported in several cancers. However, the role and underlying mechanism of miR-106 in colorectal cancer (CRC) have not been addressed.

**Methods:**

Quantitative RT-PCR(qRT-PCR) was performed to evaluate miR-106b levels in CRC cell lines and patient specimens. Cell proliferation was detected using MTT assay, and cell migration and invasion ability were evaluated by wound healing assay and transwell assay. The target gene of miR-106b was determined by qRT-PCR, western blot and luciferase assays.

**Results:**

miR-106b was significantly up-regulated in metastatic CRC tissues and cell lines, and high miR-106b expression was associated with lymph node metastasis and advanced clinical stage. In addition, miR-106b overexpression enhances, whereas miR-106b depletion reduces CRC cell migration and invasion. Moreover, we identify DLC1 as a direct target of miR-106b, reveal its expression to be inversely correlated with miR-106b in CRC samples and show that its re-introduction reverses miR-106b-induced CRC cell migration and invasion. Furthermore, survival analyses showed the patients with high mi-106b/low DLC1 had shorter overall survival (OS) and disease-free survival (DFS) rates, and confirmed miR-106b may be an independent prognostic factor for OS and DFS in CRC patients.

**Conclusions:**

Our findings indicate that miR-106b promotes CRC cell migration and invasion by targeting DLC1. This miRNA may serve as a potential prognostic biomarker and therapeutic target for CRC.

## Background

Colorectal cancer (CRC) is one of the most common malignant tumors worldwide and accounts for the fifth leading cause of cancer death in China [1, 2]. Despite achievements in the treatment in the few past decades, CRC remains a major public health concern, resulting in more than 600,000 deaths each year. The high mortality rate of CRC is related mainly to frequent tumor recurrence and metastasis after surgical resection [3]. Thus, there is an urgent need for the identification of metastatic factors and understanding the molecular mechanisms underlying CRC.

MicroRNAs (miRNAs) are small (approximately 22 nucleotides in length) non-coding RNAs that recognize and bind to partially complementary sequences of their target mRNA, resulting in either mRNA degradation or inhibition of its translation [4]. MiRNAs regulate the expression of a wide variety of target genes, and are therefore involved in a wide range of biological processes including cell proliferation, development and differentiation [5–7]. Furthermore, increasing numbers of miRNAs have been observed in various types of cancer and may be involved in modulating cancer cell behaviors [8–13]. These data emphasize the importance of miRNAs in cancer development and provide new insights into understanding the molecular mechanism of tumorigenesis and cancer metastasis.

Microarray studies have identified a number of microRNAs that are up- or down-regulated in CRC, including miR-106b [14, 15]. To date, miR-106b has been found to be deregulated in some types of cancers, such as increased in bladder cancer [16], renal cell carcinoma [17], laryngeal carcinoma [18] and hepatocellular carcinoma [19], while decreased in endometrial cancer [20]. However, no specific studies have been conducted to reveal the role of miR-106b in CRC.

Hence, our study was aimed to identify the role of miR-106b in CRC. In present study, miR-106b expression was analyzed in CRC tissues and cell lines. After that, we assessed the clinical significance of miR-106b in colorectal cancer, and to investigate the effects of miR-106b on CRC cell proliferation, migration and invasion and further discuss the mechanisms of action of miR-106b by identifying its potential target gene.

## Materials and methods

### Patients and tissue samples

Surgical specimens of cancer tissue and adjacent normal mucosa (at least 5 cm from the margin of the tumor) were obtained from 95 patients with colorectal cancer who underwent surgery at The Affiliated Hospital of North Sichuan Medical College between January 2006 and March 2009. Among the 95 patients, 2 patients diagnosed with distant metastases were excluded from our study because these cases were too few for meaningful statistical analysis. None of patients had received preoperative adjuvant therapy. After collection, all tissue samples were immediately frozen in liquid nitrogen and stored at −80 °C until use. Tumor stage was classified according to the 7th edition of the UICC/AJCC TNM staging system for CRC. Informed written consent was obtained from each patient, and research protocols were approved by the Medical Ethics Committee of North Sichuan Medical College.

For follow-up, all patients were evaluated at the outpatient clinic once per 3–6 month after discharge from hospital. The median follow-up period was 61 months (range, 11–81months). Follow-up studies included laboratory analysis, physical examination and computed tomography if necessary. Patients who died from diseases other than CRC or from unexpected events were excluded in this study. Overall survival was defined as the time from surgery to death, and disease-free survival as the time from surgery to first tumor recurrence (local recurrence and/or distal metastasis). Cases were censored at the date of last follow up.

### Cell culture

The human CRC cell lines (SW480, HCT116, HT29, SW620 and LoVo), the human embryonic kidney cell line 293 T and the normal colon epithelium cell line FHC were obtained from the American Type Culture Collection. The CRC cell lines and 293 T cells were cultured in DMEM (Invitrogen, Carlsbad, CA, USA) supplemented with 10 % fetal bovine serum (FBS; Invitrogen), and FHC cells were grown in DMEM: F12 supplemented with 10 % FBS. All cells were maintained in a humidified incubator at 37 °C with 5 % CO_2_.

### Oligonucleotides and plasmid transfection

MiR-106b mimics or inhibitor (anti-miR-106b) and their negative controls (miR-nc mimics or anti-miR-nc) were obtained from RiboBio (Guangzhou, China). The open reading frame of DLC-1 that was amplified by PCR using the primers containing KpnI and EcoRI restriction sites and subcloned into vector pcDNA 3.1(+) (Invitrogen) to generate the construct pcDNA-DLC1. The primers used were 5’-CGAACGGTACCTGCTTGATGTGCAGAAAGAAGCC-3’ forward and 5’-AAGGATCCTCACCTAGATTTGGTGTCTTTG-3’ reverse. The empty vector served as a negative control. Transfection was carried out using Lipofectamine 2000 reagent (Invitrogen) according to the manufacturer’s instructions.

### RNA extraction and quantitative real-time PCR

Total RNA, including miRNA, was isolated from tissues or cell lines using TRIzol reagent (Invitrogen) according to manufacturer’s instructions. For miRNA expression analysis, reverse transcription was performed using the TaqMan microRNA reverse transcription kit (Applied Biosystems, Foster City, CA, USA). Mature miR-106b levels were quantified with TaqMan miRNA assays (Applied Biosystems). For DLC1 mRNA detection, reverse transcription was performed using the PrimeScript RT reagent Kit (Takara, Dalian, China). Quantitative PCR was performed using SYBR Premix Ex Taq (Takara) on the ABI 7500 real-time PCR System (Applied Biosystems). U6 snRNA or β-actin was used as internal control. The primer Sequences were as follows: DLC1, 5’-CCGCCTGAGCATCTACGA-3’ forward and 5’-TTCTCCGA CCACTGATTGACTA-3’ reverse; β-actin, 5’-CCAAGGCCAACCGCGAGAAGATGAC-3’ forward and 5’-AGGGTACATGGTGGTGCCGCCAG AC-3’ reverse. The relative expression levels were calculated using the 2^-ΔCT^ method, and fold changes were calculated by the equation 2^-ΔΔCT^. For the study of correlations between miR-106b and DLC1 expression, and correlations between miR-106b/DLC1 expression and survival in CRC patients, the miR-106b and DLC1 expression levels were classified to low or high group according to their respective median expression.

### Cell proliferation analysis

Cell proliferation was measured using the MTT assay. Briefly, the transfected cells were plated in 96-well plates at 5 × 10^3^ per well in a final volume of 100 μl, and 20 μl of 5 mg/ml MTT was added to each well at 24, 48, 72 and 96 h. After incubation at 37 °C for 4 h, the MTT solution was removed, and 150 μl dimethyl sulfoxide (DMSO) was added to each well followed by measuring the absorbance at 570 nm on a SpectraMax M5 microplate reader (Molecular Devices, Sunnyvale, CA, USA).

### Wound healing and matrigel invasion assays

For the wound healing assay, cells (5 × 10^5^) were seeded into six-well plates and cultured under standard conditions. When the cells reached confluence, a wound was made by scraping the cell monolayer with a 200 μl pipette tip. Cell migration was determined by measuring the movement of cells into the scraped area. Representative images (20×) of wound closure were captured at 0 h and 48 h using an inverted microscope. For the cell invasion assay, cells (1 × 10^5^) in serum-free medium were placed into the upper chamber of a 24-well Transwell Chamber (8 μm pore size, Corning Costar Corporation, Cambridge, MA, USA) coated with Matrigel (BD Biosciences, San Jose, CA, USA). The chambers were incubated for 48 h with culture medium containing 10 % FBS added to the lower chamber. The non-invaded cells were removed with cotton swabs. Cells which had invaded to the lower surface were fixed, stained and counted using an inverted microscope (20×). All experiments were performed in triplicate.

### Luciferase reporter assay

For luciferase reporter experiments, the wild-type and mutated 3’UTR of DLC1 mRNA were subcloned into the *XhoI* and *NotI* site of the psicheck-2 vector (Promega, Madison, WI, USA). and the new vectors were named psicheck-2-DLC1-WT and psicheck-2-DLC1-MUT, respectively. the following primers were used to amplify specific fragments: DLC1-WT, forward 5’- CACAACTCGAGGAATCCACCCA GAAAGGGGG-3’ and reverse 5’- CACAACACAAGCGGCCGCGTG G CTCAGTTGCAGTTTGG-3’ and DLC1-MUT, forward 5’-AGCAAGTA GTGAAATTCCCTGTTAGAATTCTTTGCATTTT-3’ and reverse 5’- GG AATTTCACTACTTGCTTGATTTAAGAGTAAGTGTTATC-3’. For the luciferase reporter assay, HEK293T cells (1 × 10^5^ cells/well) were cultured in a 24-well plate and co-transfected with 40nM miR-106b mimics or inhibitor, 200 ng of psicheck-2-DLC1-WT or psicheck-2-DLC1-MUT, and 2 ng of pRL-TK (Promega) by using Lipofectamine 2000. The pRL-TK vector was used as a normalisation control. After transfection for 48 h, cells were harvested and assayed with Dual- Luciferase Reporter Assay System (Promega) according to the manufacturer’s protocols.

### Western blot analysis

Cultured cells were lysed with RIPA lysis buffer containing proteinase inhibitor. Proteins were separated via SDS-PAGE and transferred onto PVDF membrane. After blocking, the membrane was probed with mouse anti-DLC1 (1:500; Santa Cruz Biotechnology, Santa Cruz, CA, USA) or mouse anti-GAPDH (1:1000; Santa Cruz) overnight at 4 °C, followed by incubation with HRP-conjugated secondary antibody (Santa Cruz). Signals were visualized using ECL regents (Millipore, MA, USA).

### Statistical analysis

For continuous variables, data are expressed as mean ± standard deviation (SD). Statistical significance between groups was analyzed by Student’s *t*-test, Mann–Whitney *U* test or Kruskal-Wallis test, as appropriate. The postoperative survival rate was analyzed with Kaplan–Meier method and the survival differences of patient subgroups were compared by the log-rank test. A Cox proportional hazards model was used for multivariate analysis. The correlation between miR-106b and DLC1 was determined by Chi-squared test and Spearman’s correlation analysis. Statistical analyses were conducted using IBM SPSS Statistics (Version 19, IBM SPSS, Chicago, IL, USA). *P* < 0.05 was considered significant difference.

## Results

### MiR-106b is upregulated in metastatic CRC tissues and cell lines

**Fig. 1:**
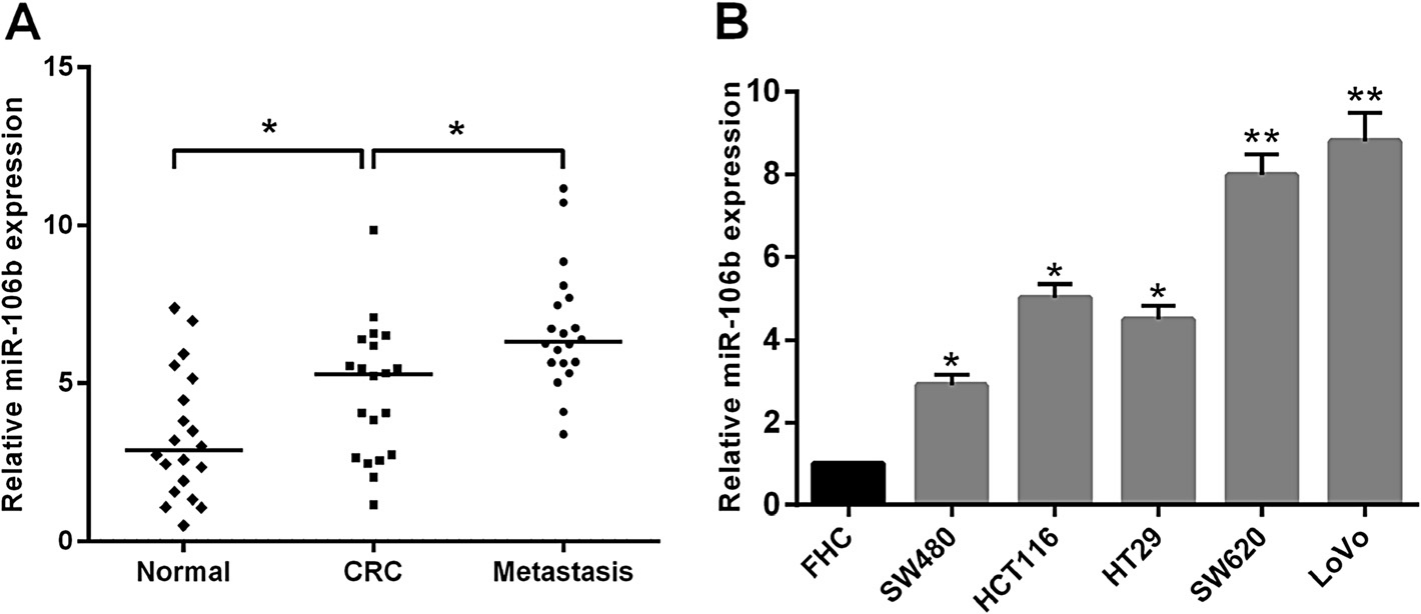
The relative expression levels of miR-106b in CRC tissues and cell lines. **a** The relative expression of miR-106b in matched normal tissues, primary CRC tissues and lymph node metastatic tissues. The expression of miR-106b was quantified by qRT-PCR and normalized to U6 snRNA; *P* values were calculated using the paired *t*-test. **b** The relative expression of miR-106b in five CRC cell lines (HT29, HCT116, SW480, SW620 and LoVo) and normal colonic cell line (FHC). *P < 0.05, **P < 0.01

In this study, the levels of miR-106b were first measured by quantitative real-time PCR (qRT-PCR) in 20 pairs of normal tissues, primary CRC tissues and lymph node metastatic tissues. As shown in Fig. 1a, miR-106b expression was significantly increased in primary CRC tissues compared to their matched normal tissues. Furthermore, in comparison to primary CRC tissues, miR-106b levels were significantly higher in lymph node metastatic tissues (*P* < 0.05, Fig. 1a). Consistent with these observations, the expression of miR-106b was significantly up-regulated in all five CRC cell lines compared with the normal colonic cell line (Fig. 1b). Also, among the five CRC cell lines, miR-106b levels were higher in metastatic CRC cells (SW620 and LoVo) compared with non-metastatic ones (HT29, HCT116 and SW480) (Fig. 1b). These findings suggest that up-regulation of miR-106b might play a role in CRC metastasis.

### Correlation of miR-106b with clinicopathological factors of colorectal cancer patients

**Fig. 2:**
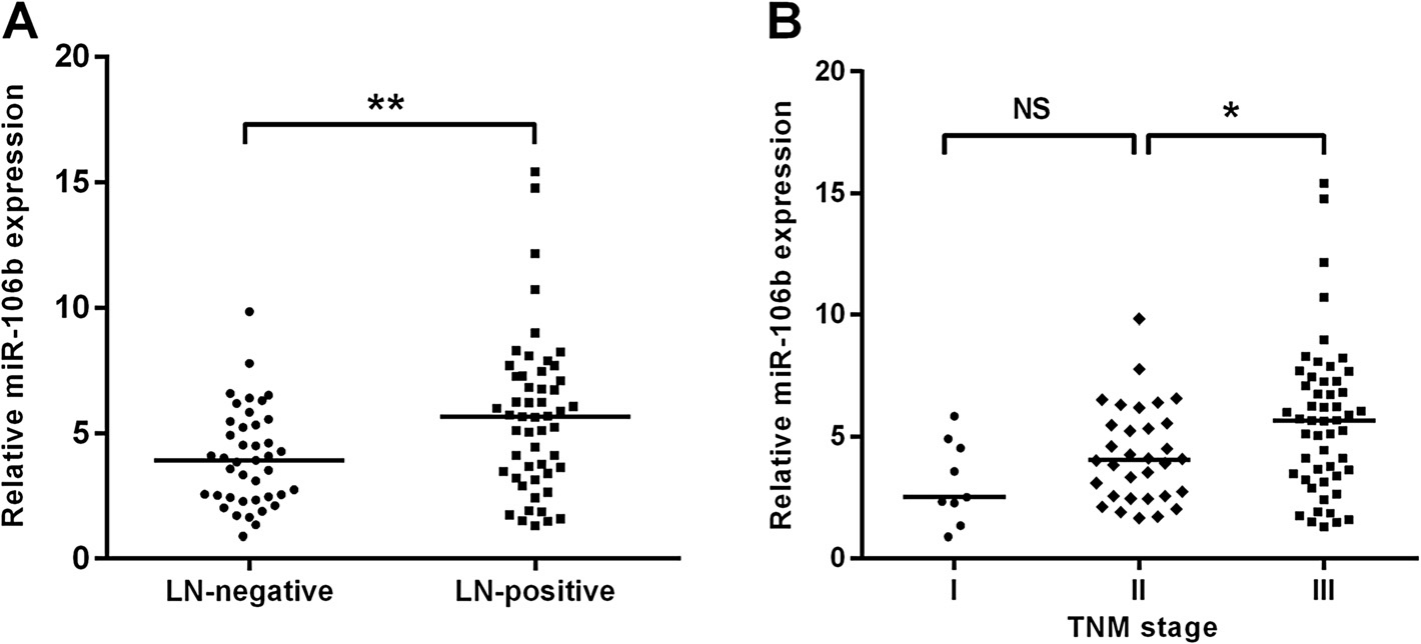
Upregulation of miR-106b is associated with advanced clinical stage and positive lymph node metastasis in CRC patients. **a** High expression of miR-106b was associated with lymph node metastasis; patients were classified into lymph node metastasis negative group (LN-negative) and positive group (LN-positive). **b**The expression levels of miR-106b in different clinical stage of CRC patients. The expression of miR-106b was quantified by qRT-PCR and normalized to U6 RNA. *P < 0.05, **P < 0.01, NS: non- significant

To further investigate the clinicopathological significance of miR-106b, we measured miR-106b expression by qRT-PCR in a larger cohort of CRC tissues (n = 93). As shown in Table 1, the miR-106b expression levels in the tissues of CRC patients with lymph node metastasis were significantly higher compared with those without metastasis (*P* = 0.003, Fig. 2a). In addition, patients with advanced stage(stage III) had higher miR-106b expression than patients with early stage (stage I and II) (*P* = 0.008, Fig. 2b). However, no significant associations were found between miR-106b expression and gender, age, tumor location, tumor size, tumor depth and differentiation.

**Table 1:** Association of miR-106b expression with clinicopathologic factors of colorectal cancer patients

Variables	n	miR-106b expresion	*P*
Gender			0.422
Male	54	4.619 ± 2.616	
Female	39	5.102 ± 3.158	
Age(years)			0.609
<60	43	4.657 ± 2.652	
≥60	50	4.962 ± 3.028	
Tumor Location			0.243
Colon	41	5.212 ± 3.176	
Rectum	52	4.514 ± 2.553	
Tumor size(cm)			0.341
≤5	59	4.607 ± 2.543	
>5	34	5.194 ± 3.323	
Tumor depth			0.106
T1-T2	27	4.053 ± 1.997	
T3-t4	66	5.114 ± 3.115	
Differentiation			0.516
Well	19	4.148 ± 2.055	
Moderate	48	4.784 ± 2.851	
Poor	26	5.388 ± 3.298	
Lymph node metastasis			0.003
Absent	41	4.015 ± 1.940	
Present	52	5.679 ± 3.096	
TNM stage			
I	9	3.219 ± 1.685	0.008
II	32	4.264 ± 1.957	
III	52	5.679 ± 3.096

### MiR-106b Promotes migration and invasion of CRC cells

**Fig. 3:**
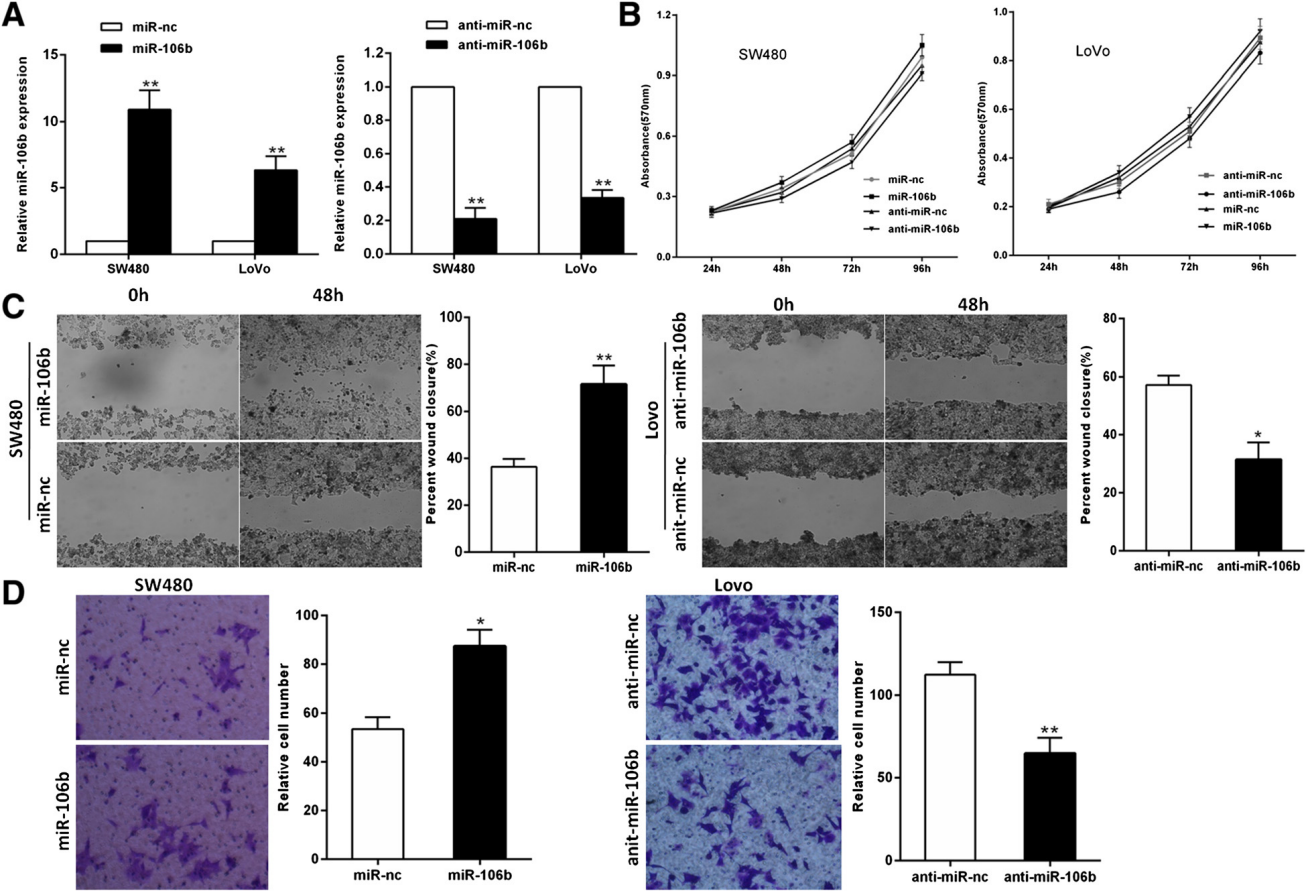
miR-106b promotes CRC cell migration and invasion. **a** The expression levels of miR-106b were tested by qRT-PCR in CRC cells transfected with miR-106b, anti-miR-106b, and their respective negative controls. **b** The effect of miR-106b expression on cell proliferation was measured by MTT assay. **c** The effect of miR-106b expression on migration of CRC cells using wound scratch healing assay. **d** The effect of miR-106b expression on invasion of CRC cells using transwell invasion assay. *P < 0.05, **P < 0.01

To explore the potential biological function of miR-106b in CRC progression, we transiently modulated the miR-106b expression level by transfection with miR-106b mimics or inhibitor. Re-expression or inhibition of miR-106b was confirmed by qRT-PCR (Fig. 3a). Intriguingly, the miR-106b expression had no effect on both types of CRC cell proliferations (Fig. 3b). We then assessed the effect of miR-106b on the migratory and invasive capacity of CRC cells using the wound-healing assay and matrigel invasion assay. As shown in Fig. 3c and D, miR-106b overexpression significantly promoted the migration and invasion of SW480 cells. In contrast, miR-106b knockdown suppressed LoVo cell migration and invasion. These observations demonstrate that miR-106b significantly promotes migration and invasion of CRC cells.

### MiR-106b directly targets DLC-1 in CRC cells

**Fig. 4:**
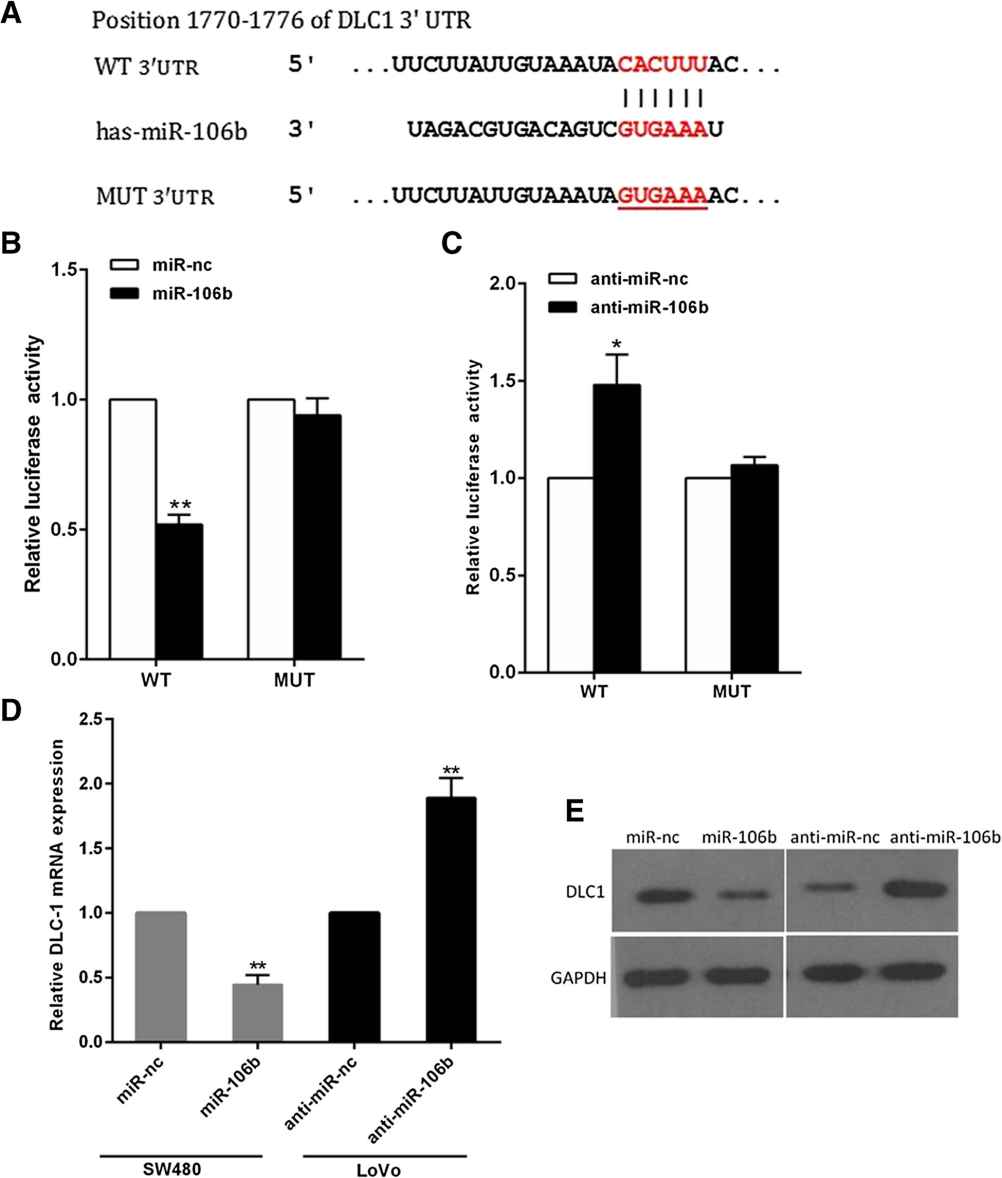
DLC1 is a direct target of miR-29c. **a** The wild-type and mutant of putative miR-106b target sequences of DLC1 3’UTR. (**b,c**) Analysis of the luciferase activity of psicheck-2-DLC1 3’UTR WT and MUT vector in HEK293T cells by miR-106b or anti-miR-106b. **d** The DLC1 mRNA levels in the indicated cells was analyzed by qRT-PCR. **e** The DLC1 protein levels in the indicated cells were examined by western blot. *P < 0.05, **P < 0.01

To characterize the mechanism by which miR-106b promotes cell migration and invasion, we searched for potential target genes of miR-106b using three publicly available databases, TargetScan, Pictar and miRanda. All of the algorithms indicated that DLC1 was a theoretical target of miR-106b(Fig. 4a).

DLC-1 was identified and selected for further analysis, as it is a metastasis-suppressor gene which is down-regulated in several tumor types [21]. To confirm whether or not DLC-1 was a direct target of miR-106b, we inserted wild-type or mutant 3’UTR sequences immediately downstream of the luciferase reporter gene and co-expressed these with either miR-106b or anti-miR-106b in HEK293 cells. As shown in Fig. 4b, miR-106b overexpression caused a clear decrease in relative luciferase activity, whereas miR-106b silencing increased the luciferase activity (Fig. 4c). In addition, mutation of the binding site of miR-106b in the 3’UTR of DLC-1 abolished both the effect of miR-106b and anti-miR-106b (Fig. 4b,c), confirming that miR-106b can bind to the DLC-1 3’UTR. Furthermore, qRT-PCR and western blotting analyses showed that miR-106b overexpression significantly reduced the levels of DLC1 mRNA and protein in SW480 cells, while miR-106b knockdown increased DLC1 levels (Fig. 4d,e). Together, these results strongly support a direct suppression of DLC1 by miR-106b by means of mRNA degradation as well as translational repression.

### DLC1 mediates miR-106b-induced migration and invasion in CRC cells

**Fig. 5:**
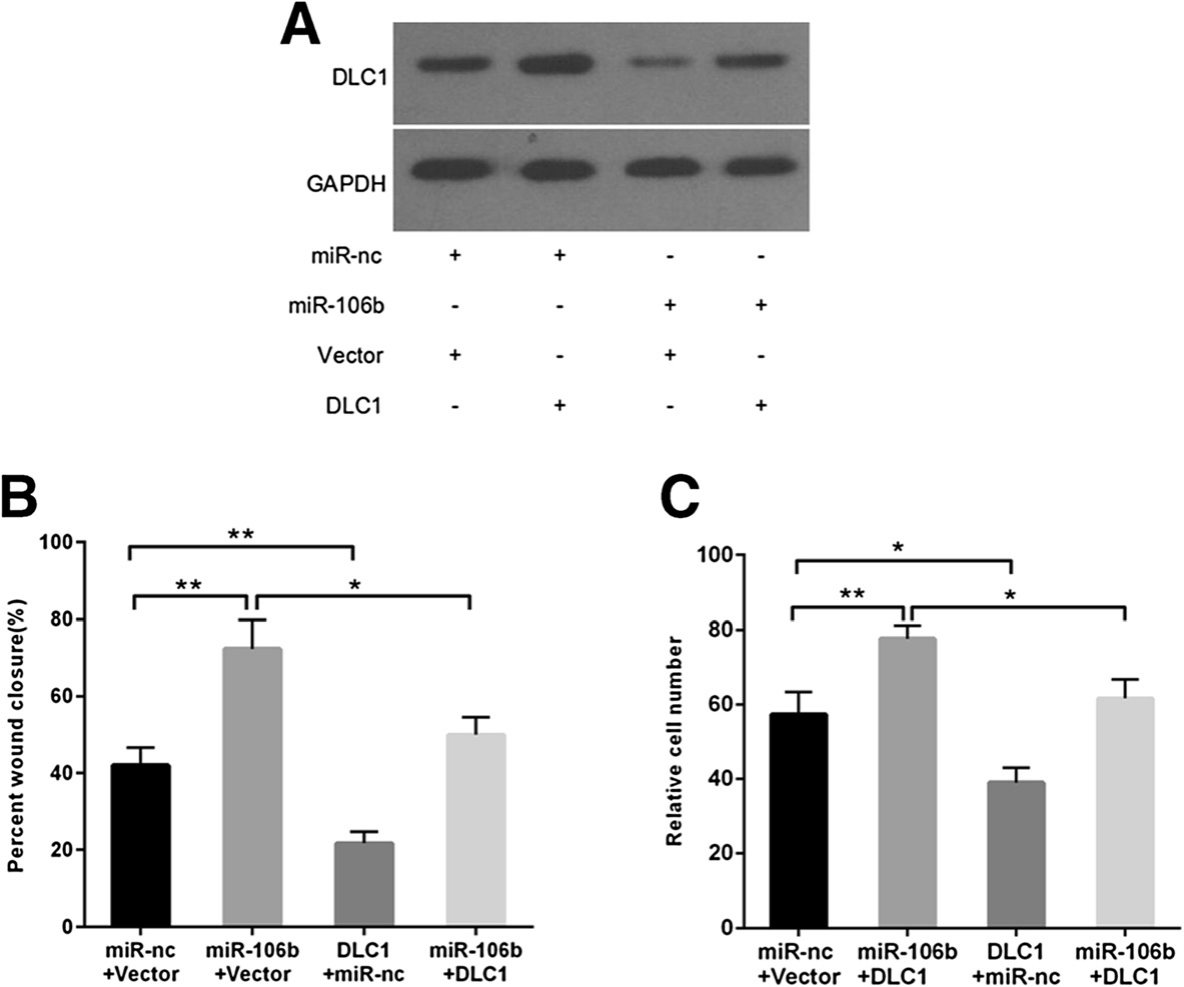
DLC1 mediates miR-106b-induced migration and invasion in SW480 cells. **a** The DLC1 protein levels were analyzed by Western blot in SW480 cells transfected with miR-106b mimic (miR-106b) and 3’UTR-negative DLC1 expression plasmid (DLC1). **b** The migratory ability was detected by the wound healing assay. **c** The invasive ability was examined by the matrigel invasion assay.*P < 0.05, **P < 0.01

To further confirm whether miR-106b promotes migration and invasion of CRC cells through DLC1, we performed a rescue experiment by introducing pcDNA3.1-DLC1 plasmid without 3’-UTR or empty vector in the presence or absence of ectopic miR-106b expression in SW480 cells. After co-transfection, the expression of DLC1 was confirmed by Western blotting as described in Fig. 5a. In agreement with the expression of target proteins, miR-106b mimics could augment the migratory and invasive ability of SW480 cells, and the decreased metastatic potential was also observed in DLC1-overexpressing cells compared with control cells (Fig. 5b, c). Furthermore, concomitant overexpression of miR-106b and DLC1 could partially abrogate miR-106b-induced migration and invasion in SW480 cells (Fig. 5b, c). Thus, these findings show that DLC1 is a functional target of miR-106b.

### Inverse correlation between miR-106b and DLC1 in CRC tissues

**Fig. 6:**
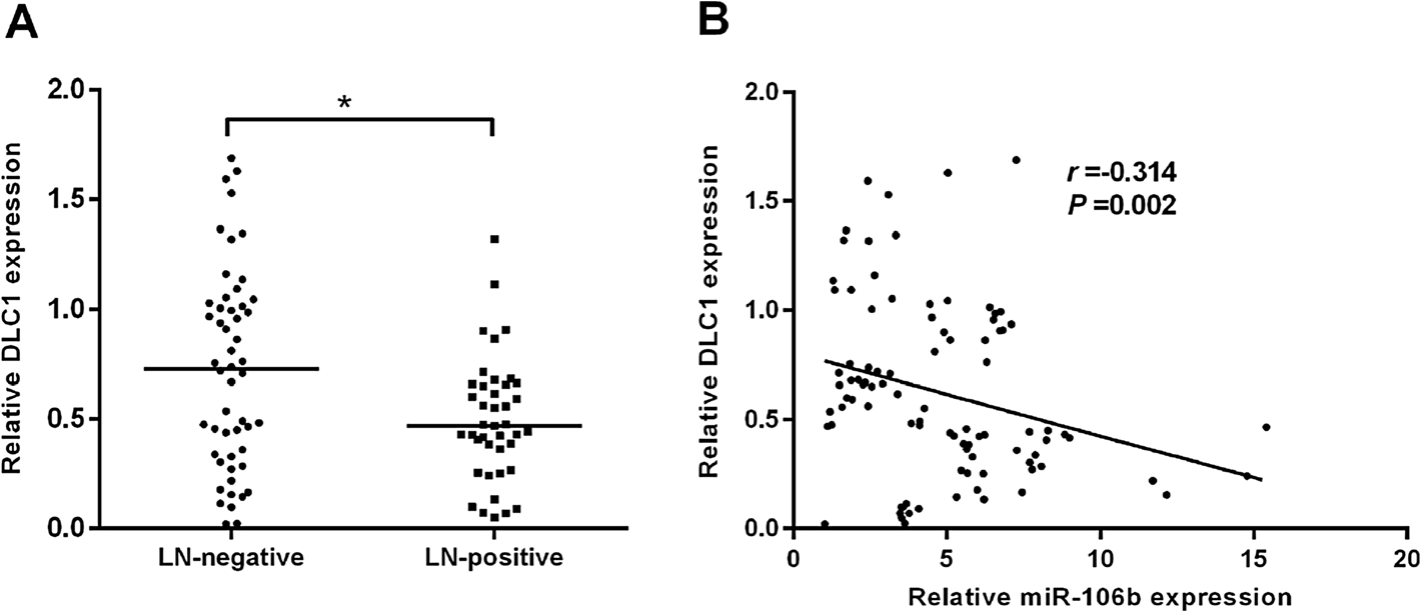
Inverse correlation between miR-106b and DLC1 in CRC tissues. **a** The expression of DLC1 in primary CRC tissues with or without lymph node metastasis by qRT-PCR (n = 93). **b** Inverse correlation between miR-106b and DLC1 in CRC tissues determined by Spearman’s correlation analysis (*r* = −0.314, *P* = 0.002). *P < 0.05

To further investigate whether miR-106b-induced modulation of DLC1 is of clinical relevance, we assessed the expression levels of DLC1 in clinical CRC tissues. As shown in Fig. 6a, DLC1 levels were lower in CRC tissues with lymph node metastasis compared with the lymph-node-negative primary CRC tissues (*P* < 0.05). We then correlated DLC1 with miR-106b expression in the same CRC specimens. A statistically significant inverse correlation was observed between mRNA levels of DLC1 and miR-106b (*P* = 0.002, Fig. 6b). Furthermore, we found that the high (or low) levels of miR-106b were more likely to be observed in CRC tissues with low (or high) levels of DLC1, providing more evidence for miR-106b mediated DLC1 regulation (*P* = 0.017, Table 2).

**Table 2:** Correlation between the expression of miR-106b and DLC-1 in 93 CRC patients

	miR-106b		n	P	Spearman’ correlation	
	low	high			*P*	*r*
	(n = 46)	(n = 47)				
DLC-1			93	0.017	0.017	−0.247
Low (n = 46)	17	29				
High (n = 47)	29	18				

### Association of miR-106b and DLC1 expression with overall and disease-free survival in CRC patients

**Fig. 7:**
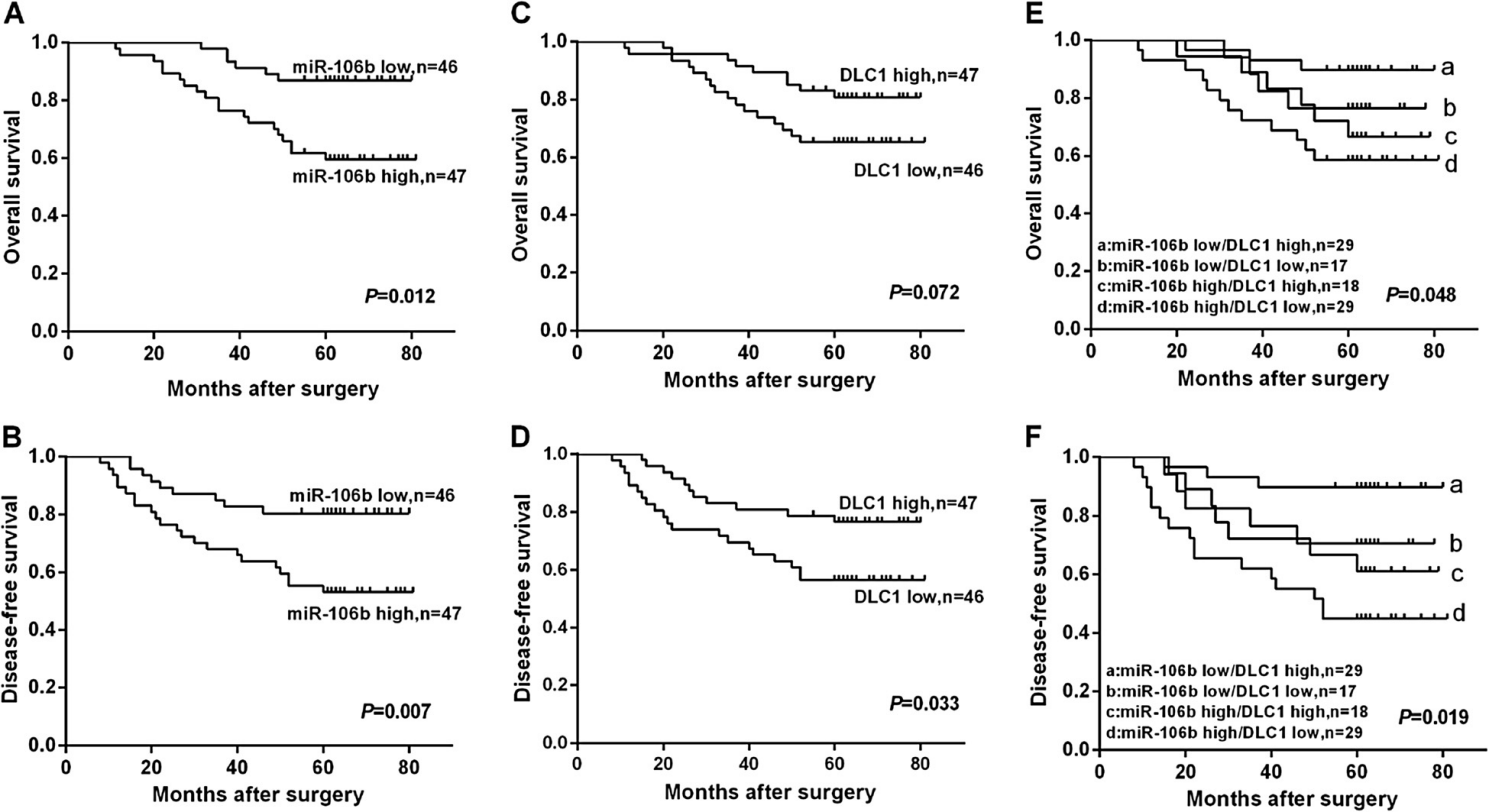
Kaplan-Meier survival curves for CRC patients. (**a**,**b**) The OS and DFS curves for CRC patients with high or low miR-106b expression. (**c**,**d**) The OS and DFS curves for CRC patients with high or low DLC1 expression. (**e**,**f**) The OS and DFS curves for CRC patients with concomitant miR-106b and DLC1 expression

Because miR-106b upregulation and DLC1 downregulation were associated with CRC metastasis, we hypothesized that miR-106b/DLC1 expression might be a prognostic factor for survival in CRC patients. To validate this hypothesis, the postoperative survival rates were analyzed using the Kaplan–Meier method and log-rank test. The results showed that patients with high miR-106b expression had shorter overall survival (OS) and disease-free survival (DFS) than those with low miR-106b expression (*P* = 0.012 for OS, Fig. 7a; *P* = 0.007 for DFS, Fig. 7b). We also observed that low DLC1 expression was associated with poor DFS (*P* = 0.033, Fig. 7d). however, low DLC1 expression was not correlated with OS (*P* = 0.072, Fig. 7c). In addition, the association between conjoined expression status of miR-106b/DLC1 and the prognosis of CRC patients was also tested. As expected, miR-106b-high/DLC1-low patients had the poorest OS and DFS in all four groups (Fig. 7e,f). These data reveal that miR-106b-DLC1 inverse regulation was associated with a poor OS and DFS.

Furthermore, Cox multivariate analysis was performed to identify independent prognostic markers for OS and DFS. Since TNM stage is determined by tumor depth and lymph node metastasis, it was not further enrolled into the multivariate analysis in this study. The results confirmed that miR-106b expression and lymph node metastasis were independent prognostic factors for OS and DFS, indicating that miR-106b could be used as biomarkers of early recurrence and poor prognosis of CRC (Table 3).

**Table 3:** Multivariate COX proportional hazards regression model analysis for overall survival and disease-free survival in CRC patients

Variables	OS			DFS		
	HR	95 % CI	*P*	HR	95 % CI	*P*
Gender	0.481	0.156-1.482	0.202	0.701	0.285-1.723	0.438
Age(years)	0.740	0.295-1.852	0.520	0.846	0.384-1.863	0.678
Tumor Location	0.482	0.133-0.749	0.267	0.891	0.388-2.046	0.786
Tumor size(cm)	0.476	0.184-1.230	0.125	0.805	0.327-1.977	0.636
Tumor depth	0.741	0.301-1.824	0.514	0.625	0.277-1.414	0.259
Differentiation	0.687	0.271-1.740	0.428	0.632	0.253-1.580	0.326
Lymph node metastasis	6.180	1.629-23.441	0.007	3.813	1.340-10.852	0.012
MiR-106b expression	3.946	1.052-14.804	0.042	3.472	1.134-10.631	0.029
DLC1 expression	0.603	0.219-1.657	0.326	0.445	1.177-1.117	0.085

## Discussion

The diagnosis and treatment of colorectal cancer (CRC) have evolved substantially during the past decade with the advent of molecular markers [22]. Recent evidence has indicated that specific miRNAs have important roles in carcinogenesis and metastasis [23], and the identification of tumor-related miRNAs and their direct target genes is critical for understanding the biological significance of miRNAs in CRC development and metastasis, and may reveal novel prognostic and therapeutic targets for CRC patients.

Previous reports revealed that miR-106b expression was high in CRC by miRNA microarray analysis [14, 15]. In the current study, we further confirmed that miR-106b was significantly up-regulated in metastatic CRC tissues, and high miR-106b expression was associated with lymph node metastasis and advanced clinical stage. These results indicate that miR-106b may play an important role in the development and progression of CRC, especially in the processes of metastasis. Several studies support our results. For example, miR-106b promotes cell migration and metastasis in hepatocellular carcinoma [24]. MiR-106b is also found to promote gastric cancer cell migration and invasion by targeting PTEN [25]. However, miR-106b has been reported to exert a metastasis-suppressor function in endometrial cancer and breast cancer [20, 26]. The discrepancies in miR-106b’s functions in different types of cancer may reflect the differences of cellular context or alternatively the targeted genes.

It has been shown that high expression of miR-106b associated with aggressive tumor phenotypes in this report. However, the biological functions of miR-106b in CRC are still unclear and need to be further elucidated. In the present study, we demonstrated that miR-106b expression was significantly increased in CRC cell lines compared with the normal colonic cell line, and CRC cell lines with metastasis capacity expressed higher miR-106b than those without metastasis. These data led us to ask whether miR-106b could regulate CRC metastasis or not. Then, we further performed gain-of-function assays in SW480 cell line (low-miR-106b) and loss-of-function assays in LoVo cell line (high-miR-106b), and found that up-regulation of miR-106b promotes migration and invasion of SW480 cells, while down-regulation of miR-106b inhibits migration and invasion of LoVo cells without affecting cell proliferation. These results indicate that miR-106b is a metastatic promoter in CRC.

To explore the mechanisms underlying the promotion of CRC cell migration and invasion mediated by miR-106b, we next set out to identify the potential target genes of miR-106b. In a number of cancers, miRNAs regulate cell proliferation and metastasis by targeting deleted in liver cancer-1 (DLC1) [27, 28]. DLC1, a member of RhoGTPase activating protein (GAP) family, has been frequently under-expressed in a wide variety of human tumors including CRC [29–31]. DLC1 is also known to have suppressive activities in tumorigenicity and cancer metastasis [32, 33]. A previous study has reported restoration of DLC1 gene inhibits proliferation and migration of human colon cells [34]. It is well known that Focal adhesion kinase (FAK) mediates several biological functions including tumor cell proliferation, migration and invasion [35]. FAK is highly expressed in CRC metastases and is activated by its phosphorylation sites thereby interacting with other signals to promote cell migration [36, 37]. A recent report has suggested that DLC1 binds to FAK and is important for its tumor suppressive function [38]. The majority of sporadic forms of colorectal cancer are characterized by deregulation of Wnt/β-catenin signaling resulting in increased transcriptional activity of the protein β-catenin [39]. It was reported that DLC1 inhibited the growth and invasion of colon cancer cells through the Wnt/β-catenin signaling pathway by upregulating GSK-3β, and downregulating β-catenin [40].

In this report, we identified DLC1 as a novel, direct target of miR-106b using luciferase reporter assays. This observation was confirmed by the fact that miR-106b overexpression diminished but miR-106b knockdown increased DLC1 mRNA and protein expression in CRC cells. Moreover, we observed that the expression of miR-106b correlated inversely with the expression of DLC1 in human CRC tissues, and ectopic expression of DLC1 significantly attenuated miR-106b induced cell migration and invasion. These results demonstrate for the first time that miR-106b can promote CRC migration and invasion by directly targeting its target gene DLC1.

A recent study reported that low DLC1 by itself did not have prognosis value in colon cancer patients, but there is a prognostic significance when low DLC1 was combined with low p15 or high Cdk6 in colon cancer patients [29–31]. In line with these findings, we found that low DLC1 expression was not associated with OS in CRC patients. However, we determined low DLC1 expression was associated with worse DFS. Moreover, miR-106b-high/DLC1-low status was significantly associated with a shorter OS and a shorter DFS. Cox multivariate analysis suggested that miR-106b represented an independent prognostic factor for both OS and DFS. On the basis of these findings, we suggest that a combined analysis of miR-106b and DLC1 expression status may enhance our accuracy in identifying patients at high risk of poor prognosis, and hence provide useful information for clinical management. The miR-106b expression may be useful as a prognostic marker for the prediction of survival and relapse in CRC patients.

In conclusion, this study demonstrates that miR-106b can significantly promote CRC cell migration and invasion by directly targeting DLC1, and revealed that high miR-106b expression could serve as an independent predictor of poor prognosis and recurrence in CRC patients. The newly identified miR-106b/DLC1 axis helps to further elucidate the complex molecular mechanisms which regulate metastasis and progression in CRC, and represents a novel strategy for prognostic prediction and the treatment of patients with CRC.
